# The effect of parietal glutamate/GABA balance on test anxiety levels in early childhood in a cross-sectional and longitudinal study

**DOI:** 10.1093/cercor/bhab412

**Published:** 2021-12-29

**Authors:** George Zacharopoulos, Francesco Sella, Kathrin Cohen Kadosh, Uzay Emir, Roi Cohen Kadosh

**Affiliations:** Department of Experimental Psychology, Wellcome Centre for Integrative Neuroimaging, University of Oxford, Oxford, OX2 6GG, UK; School of Psychology, Swansea University, Swansea, SA2 8PP, UK; Centre for Mathematical Cognition, Loughborough University, Loughborough, LE11 3TU, UK; Department of Experimental Psychology, Wellcome Centre for Integrative Neuroimaging, University of Oxford, Oxford, OX2 6GG, UK; School of Psychology, University of Surrey, Guildford, GU2 7XH, UK; School of Health Sciences, College of Health and Human Sciences, Purdue University, West Lafayette, IN 47907-2051, USA; Department of Experimental Psychology, Wellcome Centre for Integrative Neuroimaging, University of Oxford, Oxford, OX2 6GG, UK; School of Psychology, University of Surrey, Guildford, GU2 7XH, UK

**Keywords:** development, glutamate/GABA balance, magnetic resonance spectroscopy, test anxiety

## Abstract

The increased prevalence of test anxiety in our competitive society makes it a health issue of public concern. However, its neurobiological basis, especially during the years of formal education, is currently scant. Previous research has highlighted the association between neural excitation/inhibition balance and psychopathology and disease. We examined whether the glutamate/GABA profile tracks test anxiety levels in development, using a cross-sectional and longitudinal design in a cohort spanning from early childhood to early adulthood (*N* = 289), reassessed approximately 21 months later (*N* = 194). We used magnetic resonance spectroscopy to noninvasively quantify glutamate and gamma-Aminobutyric acid (GABA) levels in the intraparietal sulcus (IPS) and the middle frontal gyrus. We show that the glutamate/GABA balance within the IPS relates to current individual variation in test anxiety levels and predict future test anxiety approximately 21 months later. Critically, this relationship was observed during early childhood but not during the later developmental stages. Our results extend the use of the excitation/inhibition balance framework to characterize the psychopathology mechanisms of test anxiety, an underexplored yet widespread and debilitating condition that can impact early child development. Our findings provide a better understanding of the neurotransmitter basis underlying the emergence of anxiety disorders during development.

## Introduction

Anxiety disorders are a class of mental-health disorders characterized by excessive worry, which typically cause clinically significant distress or impairments in social, occupational, or other vital areas of functioning (American Psychiatric Association 2013). Test anxiety (henceforth TA) is aroused by the event or prospect of taking a test or examination and is characterized by a negative emotional response when facing test-related situations (Colman 2015). High TA has a debilitating effect on learning and academic performance, including standardized tests, university entrance exams, and grade point average (Mandler and Sarason 1952; Sarason and Mandler 1952; Hembree 1988; Bedell and Marlowe 1995; von der Embse et al. 2018). Moreover, the negative consequences of TA extend beyond the academic setting, including fear of negative evaluation, low self-esteem, blame assignment, and subsequent risk for anxiety and depression (Hembree 1988; Leadbeater et al. 2012). Furthermore, TA’s prevalence is increasing due to the pressure and demands of increased testing (McDonald 2001). Therefore, there is a pressing need for characterizing the underlying roots that give rise to TA during human development (Allen et al. 2020).

Our knowledge of the sociocognitive etiology of TA is rich and concerns factors such as cognitive ability, gender, school grade level, and birth order (Hembree 1988). However, our understanding of the neural underpinnings of TA is still sparse. A recent study on adults examined the neural regions involved in extended periods of elevated state anxiety (Balderston et al. 2017). It was found that the intraparietal sulcus (IPS) exhibited higher activity and more global brain connectivity when participants experienced extended periods of threat in response to receiving unpredictable electric shocks. This increased IPS activity could be derived from earlier modulation in neurotransmitters that are associated with neuroplasticity, such as GABA and glutamate (Werker and Hensch 2015). Taken together, these findings raise the possibility that IPS is a central hub for TA, and it was the case in the context of maths anxiety in children (Young et al. 2012; Hartwright et al. 2018). Since no previous studies have established a top-down modulation of IPS on the amygdala, this set of findings suggests that TA is unlikely to be underpinned by the amygdala-based canonical fear conditioning network. Instead, state anxiety was shown to be underpinned by the frontoparietal network, in which the IPS and the dorsolateral prefrontal cortex are key seeds (Arsalidou and Taylor 2011; Moeller et al. 2015; Balderston et al. 2017; Arsalidou et al. 2018; Hartwright et al. 2018; Popescu et al. 2019). Furthermore, the well-documented involvement of the frontoparietal regions in cognition (Dumontheil and Klingberg 2011; Kaufmann et al. 2011; Menon 2016; Sokolowski et al. 2017) makes them excellent candidates for the investigation of TA. For example, cognitive control ability, which is subserved by frontoparietal regions, is especially relevant to the successful execution of test-related activities and thus particularly relevant for TA.

In recent years, a framework has been centered on the idea that brain excitation/inhibition imbalance can give rise to pathological conditions, and this framework received support from research in animals and humans (Gonçalves et al. 2017; Sohal and Rubenstein 2019; Zhang et al. 2020). Impaired excitation/inhibition balance, or excitation/inhibition imbalance, may occur due to dysregulated formation or maturation of GABAergic or glutamatergic synapses (Canitano and Pallagrosi 2017). In a recent study, it was shown that during development, knockdown of a risk gene for major mental disorders (DISC1) induced excessive GABAergic inhibition that caused the loss of excitatory glutamatergic synapses resulting in excitation/inhibition imbalance (Kang et al. 2019). Human in vivo quantification of glutamate and GABA concentrations, the brain’s major excitatory and inhibitory neurotransmitters, is typically measured with the noninvasive technology magnetic resonance spectroscopy (MRS), which allows the quantification of the excitation/inhibition balance by the glutamate concentration/GABA concentration (Cohen Kadosh et al. 2015) (but see limitations of this statement in the Discussion section). Recently, a study on trait anxiety in human adults found that GABA concentration within the ventromedial prefrontal cortex (vmPFC) was positively related to levels of trait anxiety (Delli Pizzi et al. 2016). Surprisingly, the involvement of the excitation/inhibition balance in TA is unknown.

**Figure 1:**
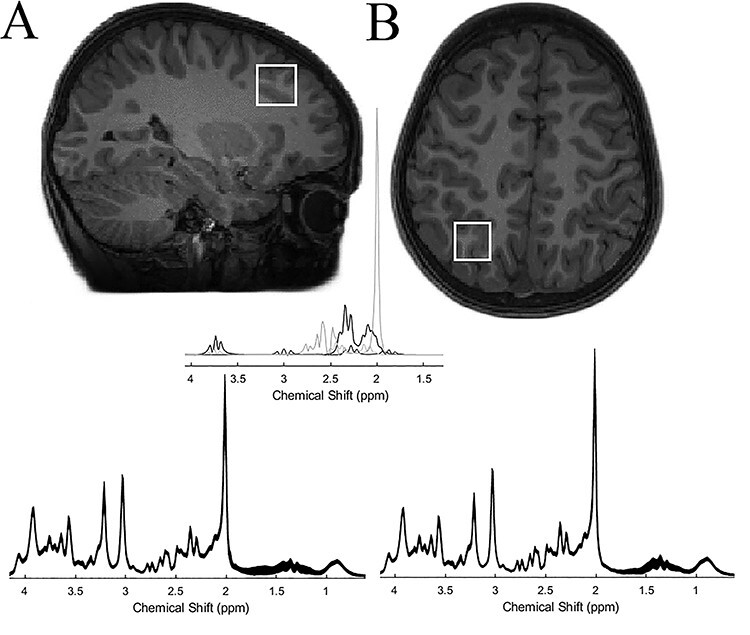
Positions of the two regions for the MRS displayed in a T1-weighted image for (*A*) MFG and (*B*) IPS are shown on sagittal and axial slices, respectively. Below each figure, the mean spectrum from our sample of each region is shown (parts per million, ppm, depicted in *x*-axis), and the thickness corresponds to ±1 SD from the mean. The middle panel shows the fit spectra of glutamate, GABA, glutamine, and N-acetylaspartate.

By combining a behavioral session and MRS to assess TA and quantify glutamate/GABA balance, respectively, the present study examined the neurochemical bases of TA in a developmental cohort spanning from early childhood to early adulthood. Based on the recent results above, we focused on two predefined central seeds of the frontoparietal network: the left IPS (Fig. 1*B*) and the middle frontal gyrus (henceforth, MFG, Fig. 1*A*). We aimed to examine the capacity of IPS and MFG glutamate/GABA balance in tracking levels of TA. Since we did not aim to interrogate the effect of brain laterality in the present study, we focused on left frontoparietal regions to keep the study duration to an acceptable length.

## Materials and Methods

### Participants

We recruited 289 participants, and the demographic information for both the first assessment and the second assessment is reported in Supplementary Material 1.

The completion of the imaging session lasted approximately 60 min, and the completion of the cognitive and behavioral tasks described here lasted approximately 30 min and was part of a larger battery of tests. All imaging data were acquired on a single scanning session during which participants watched the LEGO movie (directed by Phil et al. 2014). All participants were predominantly right-handed, as measured by the Edinburgh Handedness Inventory (Oldfield 1971) and self-reported no current or past neurological, psychiatric, or learning disability or any other conditions that might affect cognitive or brain functioning. For every assessment (i.e., first and second), adult participants received £50 compensation for their time, and children participants, depending on their age, received £25 (early childhood) and £35 (late childhood, early adolescence, and late adolescence) in Amazon or iTunes vouchers, and additional compensation for their caregiver if the participant was below 16 years old. Informed written consent was obtained from the primary caregiver and informed written assent was obtained from participants younger than 16 years old, according to approved institutional guidelines. A subset of our sample due to common attrition rate was reassessed approximately 21 months later (mean = 20.71, SD = 3.91 months). We refer to the first assessment as A1 and the second assessment as A2. The study was approved by the University of Oxford’s Medical Sciences Interdivisional Research Ethics Committee (MS-IDREC-C2_2015_016).

### MRI Data Acquisition and Preprocessing

All MRI data were acquired at the Oxford Centre for Functional MRI of the Brain (FMRIB) on a 3T Siemens (Germany) MAGNETOM Prisma MRI System equipped with a 32 channel receive-only head coil.

### Magnetic Resonance Spectroscopy

Spectra were measured by semiadiabatic localization using an adiabatic selective refocusing (semi-LASER) sequence (TE = 32 ms; TR = 3.5 s; 32 averages) (Öz and Tkáč 2011; Deelchand et al. 2015) and variable power RF pulses with optimized relaxation delays (VAPOR), water suppression, and outer volume saturation. Unsuppressed water spectra acquired from the same volume of interest were used to remove residual eddy current effects and to reconstruct the phased array spectra with MRspa (https://www.cmrr.umn.edu/downloads/mrspa/. Two 20 × 20 × 20 mm^3^ voxels of interest were manually centered in the left intraparietal sulcus (IPS) and the middle frontal gyrus (MFG) based on the individual’s T1-weighted image while the participant was lying down in the MR scanner. Acquisition time per voxel was 10–15 min, including sequence planning and shimming.

MRS neurotransmitters were quantified with LCmodel (Provencher 2001), using a basis set of simulated spectra generated based on previously reported chemical shifts and coupling constants based on a versatile simulation, pulses, and analysis (VeSPA) simulation library (Soher et al. 2011). Simulations were performed using the same RF pulses and sequence timings as in the 3T system described above. Absolute neurochemical concentrations were extracted from the spectra using a water signal as an internal concentration reference.

The exclusion criteria for data were 1) Cramér–Rao bounds (CRLB) and 2) the signal-to-noise ratio (SNR). Neurochemicals quantified with Cramér–Rao lower bounds (CRLB, the estimated error of the neurochemical quantification) >50% were classified as not detectable. We aimed to follow a relatively unbiased approach, avoid using hard threshold troubles, and follow the suggested procedure highlighted previously (Kreis 2016). In addition, since GABA concentration is relatively low (compared with glutamate), which usually induced high CRLB values, and since this study was focused on GABA and glutamate, a good compromise was to exclude CRLB >50%. Additionally, we excluded cases with an SNR beyond 3 SD (per region). We also excluded cases with a neurochemical or behavioral score beyond 3 SD (per age group), and cases where the standardized residuals in a given analysis were beyond 3 SD.

Absolute neurochemical concentrations were then scaled based on the structural properties of the selected regions and based on the predefined values shown in MRS-eq1 (see below) (Provencher 2001); these predefined scaling values were therefore determined before the data collection. To quantify the structural properties, we segmented the images into different tissue classes including gray matter (GM), white matter (WM), and cerebrospinal fluid (CSF) using the SPM12 segmentation facility. Next, we calculated the number of GM, WM, and CSF voxels within the two masks of interest separately around the left IPS and MFG in native space. Subsequently, we divided these six numbers (GM, WM, and CSF for IPS and MFG, respectively) by the total number of GM, WM, and CSF voxels to obtain the corresponding GM, WM, and CSF fraction values per participant and region. As a final computation step, we scaled the absolute neurotransmitter values to these structural fractions using the following LCmodel (Provencher 2001) computation:

\begin{eqnarray*} &&{\rm{Tissue}}\ \rm{corrected}\ \rm{concentration}\nonumber\\ &&=\left(\left(\rm{43\ 300/55\ 556}\ast \rm{GM}\ \rm{fraction}+35\ 880/55\ 556\right.\right.\nonumber\\ &&\left.\left.\ast\, \rm{WM}\ \rm{fraction}+1\ast \rm{CSF}\ \rm{fraction}\right){\rm /}\left(\rm{1-{CSF}}\ \rm{fraction}\right)\right)\nonumber\\ &&\ast\, \rm{absolute}\ \rm{neurochemical}\ \rm{concentration} \Big(\rm{MRS}-\rm{eq}1\Big) \end{eqnarray*}

This is done to perform partial-volume correction based on three tissue classes as mentioned above. The numerator is the total water concentration in the region of interest, and the factor 1/(1-CSF fraction) is the partial volume correction in that it corrects for the fact that all neurochemicals are concentrated in the gray and white matter. Glutamate concentration divided by GABA concentration was calculated for each of the two regions separately, representing a proxy measure of excitation/inhibition balance. The values 43 300, 35 880, and 55 556 are the water concentrations in mmol/L for GM, WM, and CSF, respectively, and these are the default water concentration values employed by LCModel (Provencher 2014). The numerator corrects for differing tissue water concentrations for the unsuppressed water reference, whereas the denominator corrects for the assumption that CSF is free of metabolites.

These concentration values were scaled, based on the T2 of tissue water values as can be seen in MRS-eq2. Fully relaxed unsuppressed water signals were acquired at TEs ranging from 32 to 4040 ms (TR = 15 s) to water T2 values in each region of interest (32, 42, 52, 85, 100, 115, 150, 250, 450, 850, 1650, 3250, 4040 ms). The transverse relaxation times (T2) of tissue water and percent CSF contribution to the region of interest were obtained by fitting the integrals of the unsuppressed water spectra acquired in each region of interest at different TE values with a biexponential fit (Piechnik et al. 2009), with the T2 of CSF fixed at 740 ms and three free parameters: T2 of tissue water, amplitude of tissue water, and amplitude of CSF water.

\begin{eqnarray*} &&\rm{T}2\ \rm{corrected}\ \rm{concentration}\nonumber\\ &&\quad=\rm{tissue}\ \rm{corrected}\ \rm{concentation}\nonumber\\ &&\quad\ast \exp \left(-\rm{TE}/\rm{T}2\right)\kern1.75em \Big(\rm{MRS}-\rm{eq}2\Big) \end{eqnarray*}

The results reported in the main text are derived using the quantification method of MRS-eq1. Of note, our metabolite concentration method from MRS-eq1 and MRS-eq2 was calculated based on LCmodel estimation (Provencher 2001) and was performed previously (Zacharopoulos, Emir, et al. 2021a; Zacharopoulos, Sella et al. 2021b). The results showed the same pattern across all the quantification methods.

Please note that we were able to appropriately measure GABA and separate glutamate from glutamine with our sequence. We chose to use an unedited approach since we wanted to acquire the metabolite data (not only the GABA) with the shortest possible acquisition duration to minimize possible motion artifacts during acquisition. The unedited method provided high-quality spectra from an 8 mL region of interest in 2 min. In addition, the unedited method minimizes the vulnerability of the acquisition method to system imperfections (i.e., magnetic field drift), subject motions, and magnetic field inhomogeneity. Alternatively, one can use an edited method with a larger region of interest (27 mL) and a longer acquisition duration. Because our study was focused on quantifying both GABA and glutamate, we utilized a sequence capable of detecting both of these neurotransmitters. For example, previous studies have shown that the method we employed can detect GABA in a valid and reliable manner (Barron et al. 2016; Kolasinski et al. 2017; Joers et al. 2018; Frangou et al. 2019; Hong et al. 2019; Ip et al. 2019). A study demonstrated that short-TE MRS can be employed for the reproducible detection of GABA at 3T (Near et al. 2013). Moreover, as mentioned above, we excluded cases where the CRLB was poor based on previously established criteria, thus ensuring that our quantification of single metabolites (e.g., GABA) was appropriate. Furthermore, the cross-correlation between GABA concentration and the concentration of any other metabolite in both regions was not particularly strong (i.e., it was less than 0.5) and so was the cross-correlation between glutamate and glutamine. Taken this empirical evidence together, our sequence was able to detect and quantify GABA appropriately and separate glutamate from glutamine.

### Behavioral Tests

Apart from the scanning session, participants completed a separate behavioral session to avoid fatigue.

### TA Scale

TA scale was completed by each participant who responded with a yes (coded as 1) or a no (coded as 0) response in a series of 30 questions assessing TA (Sarason et al. 1958). Two example questions are the following: “Are you afraid of school/college tests?”, “Do you worry a lot before you take a test?”. The experimenter read the questions to young children (i.e., early childhood group) at A1, and they responded verbally. A single number (mean score) was calculated for each participant where high values denoted high TA levels (Ludlow and Guida 1991). In our sample, the internal consistency as assessed with the Cronbach’s alpha was 0.88 (very high) at both the A1 and A2 separately. Across all five age groups, the TA levels at A1 were positively related to TA levels at A2 (early childhood: *r*(41) = 0.51, CI = [0.24, 0.74], *P* < 0.01; late childhood: *r*(37) = 0.44, CI = [0.09, 0.75], *P* < 0.01; early adolescence: *r*(33) = 0.73, CI = [0.54, 0.85], *P* < 0.01; late adolescence: *r*(39) = 0.76, CI = [0.60, 0.89], *P* < 0.01; early adulthood: *r*(31) = 0.81, CI = [0.63, 0.91], *P* < 0.01]).

**Figure 2:**
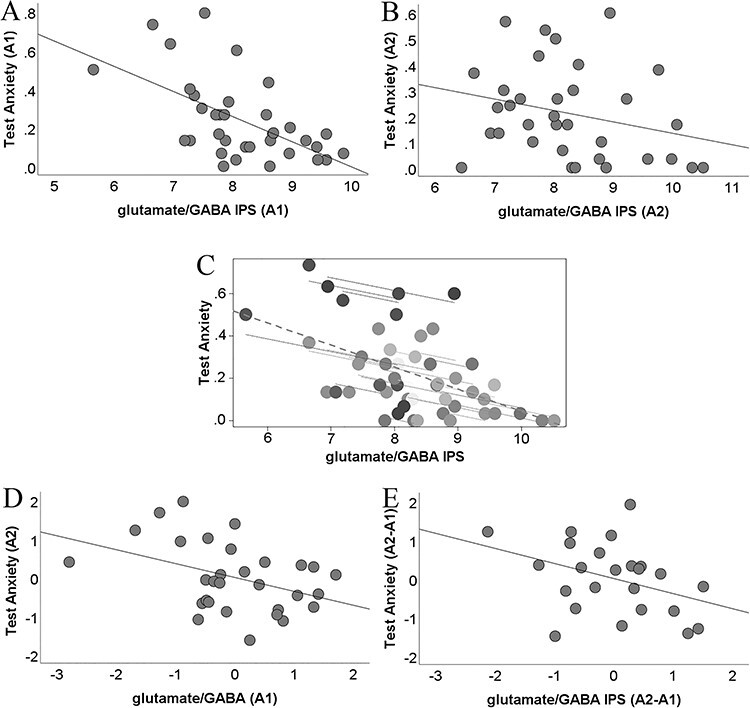
Scatterplots depicting the associations between IPS glutamate/GABA balance (micromole/g) and test anxiety in early childhood. (*A*) IPS glutamate/GABA balance at A1 was negatively related to TA at A1. (*B*) This pattern was replicated approximately 21 months later (A2) showing a negative association between the IPS glutamate/GABA balance and TA. (*C*) Repeated-measures correlation showing that within the same individual when glutamate/GABA concentration increases TA tends to decrease. Different shades of gray indicate different participants. Separate parallel lines are fit to the data from each participant. (*D*) IPS glutamate/GABA balance at A1 predicted TA approximately 21 months later (A2). (*E*) Changes in IPS glutamate/GABA balance from the second assessment to the first assessment approximately 21 months later (A2-A1) predicted changes in TA during this period. In *A*, *B*, and *C*, raw values are plotted. In *D* and *E*, the unstandardized residuals are plotted after controlling for age at A1 and age at A2, so that the gap between A1 and A2, which varies between individuals, will not impact our results.

### Maths Anxiety Assessment

Maths anxiety is a negative and debilitating emotional reaction to maths, which is defined as “a feeling of tension and anxiety that interferes with the manipulation of numbers and the solving of mathematical problems in ordinary life and academic situations^”^(Ashcraft 2002). As such, it mirrors the emotional response profile of TA, but importantly it does that for a specific domain—maths. Therefore, the addition of a math anxiety measure allowed us to use it as a covariate to interrogate whether glutamate/GABA balance can track and predict TA levels specifically. Notably, TA and maths anxiety are correlated with each other [*r*(283) = 0.62, *P* < 0.001, CI = [0.54, 0.69], at A1 and *r*(187) = 0.63, *P* < 0.001, CI = [0.52, 0.72] at A2]. Maths anxiety was assessed with the single maths anxiety (SIMA) scale (Núñez-Peña et al. 2014), whereby participants reported how maths-anxious they are on a scale from 1 to 10. As mentioned before, the experimenter read the question to young children (i.e., early childhood group) at A1, and they responded verbally.

### General Intelligence

To assess whether the brain–TA associations were confounded by the general cognitive ability, we additionally collected a measure of general cognitive ability using matrix reasoning (Wechsler 2011). The subset of matrix reasoning is a 30-item (of increasing difficulty) assessment tool that requires identifying a logical pattern in a sequence of visuospatial stimuli. The subtest is interrupted when the participant provides three consecutive wrong responses. We calculated the number of correct responses.

### Statistical Analyses

We used correlation and regression analysis. All models feature a single dependent variable (TA), and models could include one (correlation) or more (multiple regression) independent variables. Regarding the neurochemical–TA results mentioned in the main text, our alpha level was 0.05 during the first assessment analyses. To correct for multiple comparisons for the different regions (IPS and MFG) and the different age groups, we performed FDR (Benjamini and Hochberg 2000) correction at an alpha level of 0.05, which is denoted by *P*_FDR_. To confirm our hypotheses obtained from the first assessment replicated during the second assessment, we used a one-tailed test as mentioned in the Results sections. Apart from the repeated-measures correlations (see below), we always report the bootstrapped-based *P*-value (*P*), and bootstrapped-based confidence intervals (CI), derived from 5000 samples as the bootstrapping methods are not susceptible to violations of linear regression such as residual nonnormality and heteroscedasticity, which were often violated in our data (Field 2013). We defined significance based on the bootstrapped *P*-value, but for completeness, we present both *P*-values and CI in the Results section. The bootstrapped *P*-value was calculated using the following steps: 1) calculation of the standard error, SE = (Upper Bound CI − Lower Bound CI)/(2*1.95996), 2) calculation of the relevant statistic, *z* = abs(coefficient of interest/SE), and 3) calculation of the *P*-value, *P*-value = exp(−0.717*z − 0.416*z^2^). We assessed multicollinearity with variance inflation factor (VIF), but as can be seen in Supplementary Material 2, this value was always less than 10, suggesting the absence of significant levels of multicollinearity (Marquaridt 1970; Neter et al. 1989; Kennedy 2008; Hair 2009). To examine the capacity of the glutamate/GABA balance in tracking TA, we employed a multiple regression model where TA was the dependent variable, while age group and glutamate/GABA balance, as well as the interactions between age group and glutamate/GABA balance, were the independent variables. To model the age group, we recoded four dummy variables (early childhood, late childhood, early adolescence, late adolescence), each corresponding to the four age groups. Early childhood was set to 1 if the participant was from the early childhood group and 0 otherwise, late childhood was set to 1 if the participant was from the late childhood group and 0 otherwise, early adolescence was set to 1 if the participant was from the early adolescence group and 0 otherwise, and late adolescence was set to 1 if the participant was from the late adolescence group and 0 otherwise. The early adulthood group in this model was treated as the reference group to examine the changes during the different developmental stages compared with early adulthood. In the text, we present the standardized coefficients. These were obtained by z-scoring the continuous predictors and continuous dependent variables. All categorical predictors (e.g., age group or gender) were dummy-coded and were not *z*-scored. The interaction terms between the dummy-coded predictors and the continuous predictors were thus computed in the form of dummy-coded predictor* *z*-scored continuous predictor. As a first step, we run the following regression equation:

(1) \begin{eqnarray*} &&\mathrm{TA}\ \left(\mathrm{A1}\right)\sim \mathrm{glutamate}/\mathrm{GABA}\ \mathrm{balance}\ \left(\mathrm{{A}1}\right)\nonumber\\ &&\quad +\,\mathrm{early}\ \mathrm{childhood}\ \left(\mathrm{A1}\right)+\mathrm{late}\ \mathrm{childhood}\ \left(\mathrm{A1}\right)\nonumber\\ &&\quad +\,\mathrm{early}\ \mathrm{adolescence}\ \left(\mathrm{A1}\right)+\mathrm{late}\ \mathrm{adolescence}\ \left(\mathrm{A1}\right)\nonumber\\ &&\quad +\,\mathrm{early}\ \mathrm{childhood}\ \left(\mathrm{A1}\right)\ast \mathrm{glutamate}/\mathrm{GABA}\ \mathrm{balance}\nonumber\\ &&\quad \left(\mathrm{A1}\right)+\mathrm{late}\ \mathrm{childhood}\ \left(\mathrm{A1}\right)\ast \mathrm{glutamate}/\mathrm{GABA}\nonumber\\ &&\quad \ \mathrm{balance}\ \left(\mathrm{A1}\right)+\mathrm{early}\ \mathrm{adolescence}\ \left(\mathrm{A1}\right)\nonumber\\ &&\quad \ast\, \mathrm{glutamate}/\mathrm{GABA}\ \mathrm{balance}\ \left(\mathrm{A1}\right)\nonumber\\ &&\quad +\,\mathrm{late}\ \mathrm{adolescence}\ \left(\mathrm{A1}\right)\ast \mathrm{glutamate}/\nonumber\\ &&\quad \mathrm{GABA}\ \mathrm{balance}\ \left(\mathrm{A1}\right) \end{eqnarray*}

The effect of interest is the four regression slopes (i.e., of the last four interaction predictors), the significance of which assesses whether the slope in each of these four groups is significantly different from the analogous slope of the reference group (i.e., early adulthood).

Apart from examining the relation between glutamate/GABA and TA at A1 (Fig. 2*A*) and A2 (Fig. 2*B*) separately, we performed longitudinal or repeated-measures correlations, which is a robust statistical approach for combining correlations from the same participants at different measurements/conditions, and it allows longitudinal interpretation (Bakdash and Marusich 2017). Namely, a significant association between the two variables indicates that within the same individual, when one of the variables increases, the other variable increases (or decreases). The input to the statistical model for the repeated-measures correlations is the values of the variables involved at the different time-points, and a vector indicating the participant ID/number.

## Results

### Glutamate/GABA Balance Tracks TA Levels in Early Childhood

We first examined whether individual variation in glutamate/GABA balance is associated with TA levels. We found that the IPS glutamate/GABA is a significant predictor of TA during early childhood only (β = −0.5, *t*(249) = −2.01, SE = 0.22, *P* = 0.022, 95% CI = [−0.93, −0.08] (for the comparison to the other groups, see Supplementary Material 2). However, a potential limitation of our regression model is that it examines the effect of glutamate/GABA balance in each developmental stage (i.e., early childhood, late childhood, early adolescence, late adolescence) versus the reference group (early adulthood). To examine whether the significant results were not due to the arbitrary choice of the specific reference group, we re-run the regression analysis, and replicated our results, showing that the effect of glutamate/GABA balance in the early childhood group was present independently of the reference group (vs. late childhood reference group, β = −0.9, *t*(249) = −3.39, SE = 0.24, *P* < 0.001, 95% CI = [−1.36, −0.41]; vs. early adolescence reference group, β = −0.81, *t*(249) = −2.9, SE = 0.24, *P* < 0.001, 95% CI = [−1.3, −0.4]; vs. late adolescence reference group, β = −0.7, *t*(249) = −2.79, SE = 0.23, *P* = 0.002, 95% CI = [−1.18, −0.3]). Furthermore, our results held when we examined the association between the glutamate/GABA balance and TA in each of the five groups separately using correlational analysis. Again, we found a strong negative association between IPS glutamate/GABA balance and TA levels in the early childhood group only (Fig. 2*A*, *r*(33) = −0.58, CI = [−0.74, −0.36], *P* < 0.001, *P*_FDR_ = <0.001 after multiple comparison correction). Importantly, IPS glutamate/GABA tracks TA levels in early childhood even after controlling for gender (β = −0.58, *t*(32) = −4.06, SE = 0.13, *P* < 0.001, 95% CI = [−0.84, −0.32]), general cognitive ability (β = −0.57, *t*(32) = −3.87, SE = 0.14, *P* < 0.001, 95% CI = [−0.85, −0.31]), and maths anxiety (β = −0.48, *t*(31) = −3.74, SE = 0.1, *P* < 0.001, 95% CI = [−0.7, −0.26]). In addition, IPS glutamate/GABA tracks TA levels in early childhood even after we controlled for MFG glutamate/GABA, highlighting that our effect is not due to glutamate/GABA independent of the IPS (β = −0.61, *t*(29) = −3.68, SE = 0.14, *P* < 0.001, 95% CI = [−0.9, −0.35]). To examine whether our findings reflect the balance between glutamate and GABA, we assessed the effects of IPS glutamate and GABA on TA separately. The IPS glutamate and the IPS GABA were related to TA (IPS glutamate: *r*(34) = −0.34, *P* = 0.015, 95% CI = [−0.6, −0.05]; IPS GABA: *r*(33) = 0.3, *P* = 0.019, 95% CI = [0.05, 0.55]). However, the variance explained by the glutamate/GABA balance is higher compared with the variance explained by glutamate or GABA. Specifically, the amount of explained variance in the case of the balance (33%) was around three times larger than the explained variance of either the glutamate (11%) or GABA (9%). These results suggest that the balance between glutamate and GABA best tracked TA levels and provide a more parsimonious explanation.

### IPS Glutamate/GABA Balance Tracks TA Levels in Early Childhood during the Second Assessment

We then investigated whether the negative association between IPS glutamate/GABA balance and TA levels in early childhood that we detected at A1 is replicated at A2, which was indeed the case (Fig. 2*B*, *r*(32) = −0.27, 90% CI = [−0.52, 0.01], *P* = 0.045, one-tailed). Apart from examining the relation between glutamate/GABA and TA at A1 (Fig. 2*A*) and A2 (Fig. 2*B*) separately, we performed repeated-measures correlations (Bakdash and Marusich 2017), which showed that within the same individual over A1 and A2, when glutamate/GABA concentration increases TA tends to decrease [*r*(23) = −0.44, *P* = 0.03, 95% CI = [−0.72, −0.03], Fig. 2*C*].

### IPS Glutamate/GABA Balance at A1 Predicts TA Levels at A2 in Early Childhood

After establishing the association between IPS glutamate/GABA balance and TA at these two specific time windows, we examined the capacity of IPS glutamate/GABA balance to predict future TA levels in early childhood. Indeed, IPS glutamate/GABA balance at A1 predicted TA levels at A2, showing that lower glutamate/GABA balance at A1 predicts higher TA in the future [β = −0.36, *t*(26) = −2.25, SE = 0.16, *P* = 0.013, one-tailed, 90% CI = [−0.66, −0.14], Fig. 2*D*. Moreover, a decrease in IPS glutamate/GABA balance from A2 to A1 predicted an increase in TA during this period [β = −0.39, *t*(20) = −1.82, SE = 0.21, *P* = 0.038, one-tailed, 90% CI = [−0.68, 0.004], Fig. 2*E*]. For the raw neurochemical and test anxiety values at A1 and A2, see Supplementary Material 3.

## Discussion

The primary goal of the present study was to test whether the glutamate/GABA balance in key frontoparietal regions tracks current TA and predicts its future level. To this end, we quantified the glutamate/GABA balance in the left IPS and the left MFG using MRS, and we measured TA in two sessions approximately 21 months apart.

Our main finding highlights the role of the glutamate/GABA balance within the IPS, not the MFG, in tracking TA. We found that increased glutamate/GABA was associated with low levels of TA in early childhood. Furthermore, we showed that IPS glutamate/GABA balance predicted future TA in early childhood.

The effect of glutamate/GABA on TA in early childhood is in line with previous results in other domains, showing that glutamate, GABA, or the balance between them can be associated with a given cognitive function at a specific developmental stage (Cohen Kadosh et al. 2015; Zacharopoulos, Emir, et al. 2021a; Zacharopoulos, Sella et al. 2021b). For example, higher glutamate/GABA was positively associated with face processing proficiency in children but not in adults (Cohen Kadosh et al. 2015). This finding supports the hypothesis that glutamate/GABA balance is more relevant for the initial acquisition of a skill or processing, which occurs mainly during early developmental stages, rather than supporting consolidated processes, which usually occurs during later developmental stages (Cohen Kadosh et al. 2015). Future studies could examine at what particular time the involvement of glutamate/GABA balance in TA emerges, although this could be a challenging task in younger children below 6 years of age. Although it is difficult based on our study alone to exclude the possibility that TA specifically develops at 6 years of age and not before that point, still, our findings suggest that the glutamate/GABA balance becomes relevant early on when TA begins to develop. That may stem from the fact that early in development TA is more susceptible to innate biological factors and less from the environmental factors, while later in development, environmental stimulation, experience, and performance feedback may become more crucial factors in shaping TA. However, this is currently just an initial suggestion as it is equally possible that environmental factors may influence or even interact with these aforementioned biological factors shaping TA. Nonetheless, by additionally conducting repeated-measures correlations focusing on early childhood, we showed that within the same individual when glutamate/GABA increases TA tends to decrease, thus corroborating our main association on the relation between this neurochemical marker and TA.

As in our study, a previous MRS study on adults found the same direction of results between GABA levels within the vmPFC and trait anxiety, which was explained in the context of the amygdala-based canonical model of anxiety (Delli Pizzi et al. 2016). However, unlike vmPFC, a top-down modulation from IPS to amygdala has not been established, and thus, we speculate that glutamate/GABA balance within the IPS does not shape TA via the amygdala-based canonical model of anxiety. As discussed in the Introduction, unlike typical trait anxiety that is related to a sustained level of arousal that would depend on prefrontal cortex–amygdala activation, TA specifically occurs in specific settings, and this may explain why glutamate/GABA balance within the MFG was not as a relevant predictor in the early childhood group.

Previous studies combining MRS with fMRI that focused on the frontoparietal network identified an altered relationship between glutamate levels and brain activity during cognitive control in high anxious individuals. In particular, high anxious individuals did not show a positive association between prefrontal glutamate levels and prefrontal activation during cognitive control, which was present in low anxious individuals (Morgenroth et al. 2019). Given the established role of the frontoparietal network in cognitive control, “top-down” attention as well as with anxiety disorders, the finding that individual variation in trait anxiety affects the relationship between frontoparietal glutamate levels and activation suggest that anxiety could trigger ineffective task performance when cognitive control is required (Miller and Cohen 2001; Braver et al. 2009; Mochcovitch et al. 2014; Morgenroth et al. 2019). Our findings expand our knowledge on the contribution of frontoparietal network in anxiety disorders (Mochcovitch et al. 2014; Morgenroth et al. 2019) by elucidating how a specific property of the frontoparietal network (glutamate/GABA balance within one of its central hubs, the IPS) is associated with and predict anxiety in a specific developmental stage (i.e., early childhood). Given the role of IPS in frontoparietal networks supporting cognition (Dumontheil and Klingberg 2011; Kaufmann et al. 2011; Menon 2016; Sokolowski et al. 2017), and sustained anxiety (Balderston et al. 2017), our results perhaps reflect a stronger ability of young children with high glutamate/GABA concentration to exert cognitive control in general, and in test situations in particular, thus explaining reduced levels in the TA measure. Based on our results showing that glutamate/GABA balance predicts TA levels approximately 21 months later, we predict that interventions aiming at modulating the glutamate and GABA levels (Stagg et al. 2009; Sanchez-Leon et al. 2021) would induce specific alteration in TA. For example, we predict that decreasing GABA and/or increasing glutamate would decrease TA. Such studies could provide a stronger causal inference of the role of glutamate/GABA in TA. Our study found that the role of IPS glutamate/GABA on TA was particularly strong in early childhood. This can be because parietal regions are among the regions that mature earlier; therefore, the younger age group is likely to show a different excitability pattern from the other age groups (Giedd et al. 1999; Gogtay et al. 2004; Tamnes et al. 2013).

Research from animal work recently elucidated the impact of excitation/inhibition imbalance during development. Knockdown of a risk gene for major mental disorders (DISC1) significantly elevated GABA levels that decrease the maturation of glutamatergic synapses leading to overinhibition and excitation/inhibition imbalance (Kang et al. 2019). Future work should examine whether the decreased glutamate/GABA balance in participants who exhibited high TA levels may result from such genetic origins increasing GABA levels and decreasing glutamate levels.

Critically, this study has three potential limitations. First, ^1^H-MRS is not sensitive to compartmental differences in glutamate and GABA as it cannot currently distinguish between intracellular and extracellular neurotransmitter concentrations or even a portion of these based on the MRS signal alone (Dyke et al. 2017). Consequently, making direct inferences of cortical excitability/inhibition and plasticity based on the neurotransmitter concentrations derived solely from the MRS signal alone should be done with caution. Indeed several potential mechanisms have been proposed in the context of MRS concentration changes for both GABA (e.g., decreased GABA metabolism, increased catabolism, a shift of GABA into an MRS-invisible pool) and glutamate (e.g., glutamate levels were closely related to transcranial magnetic stimulation measures of local excitability; Stagg 2014). Second, even though there are observations of elevated glutamate measured with MRS when glutamate activity is up, those elevations are relatively small (typically small fractions of one percent). However, recent functional MRS work demonstrated an approximately 4% increase in glutamate during prolonged motor activation (Volovyk and Tal 2020). Therefore, taken these data together with our present findings, future studies should continue and evaluate the utility of glutamate and GABA levels as an approximate measure of excitability/inhibition balance. Third, our age-specific differences between glutamate/GABA balance within the IPS and TA could be attributed to previous experience in doing tests. Namely, the amount of testing the early childhood group has done is arguably less compared with the amount of testing of the other older age groups in our sample, and such subtle testing experience could not potentially be construed as a strong anxiety-inducing experience. However, TA at A1 and TA at A2 were positively correlated, and our findings were replicated when this cohort was older and thus had more testing-related experience; thus, we believe that our measures regarding the early childhood group even at A1 were valid in that they reflected the TA profile of each participant.

In sum, we have highlighted the role of the glutamate/GABA balance within the IPS in tracking and predicting TA during early childhood. Additional analyses established that the well-established determinants of TA did not confound our associations. These findings provide a novel understanding that could shape future studies to clarify the interplay between glutamate/GABA balance and TA to provide a better mechanistic understanding of pediatric psychopathology.

The Wellcome Centre for Integrative Neuroimaging is supported by core funding from the Wellcome Trust (203139/Z/16/Z). This work was supported by the European Research Council (Learning&Achievement 338065).

## Notes

The authors are grateful to all the participants and parents involved in this study and to Malin I. Karstens, Katarzyna Dabrowska, Laura Epton, Charlotte Hartwright, Ramona Kantschuster, Margherita Nulli, Claire Shuttleworth, and Anne Sokolich for their assistance in running this project. The authors are grateful to all Wellcome Centre for Integrative Neuroimaging (WIN) staff, in particular Nicola Filippini, Emily Hinson, Eniko Zsoldos, Caitlin O’Brien, Jon Campbell, Michael Sanders, Caroline Young, and David Parker. *Conflict of Interest*: None declared.

## Supplementary Material

Final_SM_bhab412
