# Supplementary material for: The effect of parietal glutamate/GABA balance on test anxiety levels in early childhood in a cross-sectional and longitudinal study
Source: Cereb Cortex. 2021 Dec 29;32(15):3243–53. doi: 10.1093/cercor/bhab412 (PMC9340388; doi:10.1093/cercor/bhab412)
Supplement: Final_SM_bhab412 [file final_sm_bhab412.docx]

**Supplementary Material**

**Supplementary Material 1.** Gender and mean age (standard deviation in parentheses) during the first (A1, top half) and the second (A2, bottom half) assessment.

| **Group** | **Females** | **Males** | **Age** |
| --- | --- | --- | --- |
| First Assessment (A1) | | | |
| Early Childhood | 27 | 23 | 6.5 (.29) |
| Late Childhood | 27 | 24 | 10.45 (.32) |
| Early Adolescence | 25 | 25 | 14.4 (.33) |
| Late Adolescence | 54 | 30 | 16.89 (.39) |
| Early Adulthood | 21 | 33 | 18.89 (.62) |
| Second Assessment (A2) | | | |
| Early Childhood | 22 | 21 | 8.19 (.37) |
| Late Childhood | 22 | 18 | 12.29 (.49) |
| Early Adolescence | 16 | 19 | 16.13 (.40) |
| Late Adolescence | 25 | 17 | 18.45 (.55) |
| Early Adulthood | 12 | 22 | 20.6 (.65) |

**Supplementary Material 2**

These analyses examine whether glutamate/GABA balance tracked individual variation in TA. To this end, we employed a multiple regression model where the dependent variable was TA and the independent variables were the MFG glutamate/GABA balance, age and corresponding neurotransmitter*age interactions as described in the eq1 in the **Material and** **Methods** section. Note that we obtained the main effect of MFG glutamate/GABA. However, we consider this effect as unreliable and likely to represent a type-I error, as its effect was small, and it was not replicated when we run the same model for A2 (β=0.16, t(162)=0.6, SE=0.32, P=0.3, one-tailed, 90% [-0.42, 0.60]). β=standardized regression coefficient, T=t-statistic, SE=standard error, P=bootstrapped p-value, CI L= 95% confidence intervals lower bound, CI U= 95% confidence intervals upper bound.

**Supplementary Material 2.1.** Table depicting the results of each predictor in the case of MFG in predicting TA at A1 with early adulthood as the reference group.

|  | **β** | **T** | **SE** | **P** | **CI L** | **CI U** | **VIF** |
| --- | --- | --- | --- | --- | --- | --- | --- |
| (Constant) | -0.38 | -2.53 | 0.12 | 0.0014 | -0.60 | -0.13 | NaN |
| early childhood | 0.34 | 1.23 | 0.31 | 0.2801 | -0.27 | 0.95 | 2.45 |
| late childhood | 0.12 | 0.53 | 0.21 | 0.5938 | -0.30 | 0.53 | 2.08 |
| early adolescence | 0.54 | 2.54 | 0.21 | 0.0083 | 0.15 | 0.95 | 1.80 |
| late adolescence | 0.76 | 4.03 | 0.16 | 0.0000 | 0.44 | 1.06 | 2.14 |
| glutamate/GABA MFG*early childhood | 0.02 | 0.10 | 0.20 | 0.9157 | -0.38 | 0.39 | 3.74 |
| glutamate/GABA MFG*late childhood | 0.11 | 0.43 | 0.21 | 0.6276 | -0.33 | 0.50 | 2.40 |
| glutamate/GABA MFG*early adolescence | 0.22 | 0.82 | 0.27 | 0.4264 | -0.31 | 0.73 | 1.80 |
| glutamate/GABA MFG*late adolescence | 0.21 | 1.05 | 0.15 | 0.1572 | -0.10 | 0.48 | 3.65 |
| glutamate/GABA MFG | -0.26 | -1.54 | 0.12 | 0.0353 | -0.49 | 0.00 | 7.82 |
|  | | | | | | |  |

**Supplementary Material 2.2.** Table depicting the results of each predictor in the case of IPS in predicting TA at A1 with early adulthood as the reference group.

|  | **β** | **T** | **SE** | **P** | **CI L** | **CI U** | **VIF** |
| --- | --- | --- | --- | --- | --- | --- | --- |
| (Constant) | -0.40 | -2.75 | 0.12 | 0.0012 | -0.63 | -0.15 | NaN |
| early childhood | 1.09 | 3.13 | 0.35 | 0.0022 | 0.41 | 1.81 | 4.21 |
| late childhood | -0.02 | -0.09 | 0.20 | 0.9248 | -0.40 | 0.39 | 2.12 |
| early adolescence | 0.63 | 3.05 | 0.20 | 0.0021 | 0.24 | 1.03 | 1.74 |
| late adolescence | 0.79 | 4.04 | 0.17 | <.001 | 0.46 | 1.13 | 2.45 |
| glutamate/GABA IPS*early childhood | -0.50 | -2.01 | 0.22 | 0.0220 | -0.93 | -0.08 | 5.64 |
| glutamate/GABA IPS*late childhood | 0.40 | 1.74 | 0.23 | 0.0770 | -0.06 | 0.83 | 2.38 |
| glutamate/GABA IPS*early adolescence | 0.31 | 1.27 | 0.23 | 0.1695 | -0.10 | 0.80 | 1.63 |
| glutamate/GABA IPS*late adolescence | 0.22 | 0.98 | 0.22 | 0.3259 | -0.21 | 0.64 | 2.63 |
| glutamate/GABA IPS | -0.23 | -1.53 | 0.14 | 0.0984 | -0.50 | 0.04 | 6.57 |
|  | | | | | | |  |

**Supplementary Material 3.** Raw values of IPS glutamate/GABA, IPS glutamate, IPS GABA, and test anxiety (TA) in our early childhood sample at A1 and A2**.**

| **A1** | | | | **A2** | | | |
| --- | --- | --- | --- | --- | --- | --- | --- |
| **TA** | **GLU/GABA** | **GLU** | **GABA** | **TA** | **GLU/GABA** | **GLU** | **GABA** |
| 0.33 | 7.93 | 11.97 | 1.51 | 0.30 | 8.33 | 12.34 | 1.48 |
| 0.07 | 8.95 | 14.28 | 1.60 | 0.00 | 10.33 | 13.49 | 1.31 |
| 0.50 | 5.66 | 11.78 | - | 0.17 | 8.04 | 12.13 | 1.51 |
| 0.79 | - | - | - | 0.73 | - | - | - |
| 0.30 | 7.48 | 11.18 | 1.49 | 0.03 | 8.77 | 10.51 | 1.20 |
| 0.27 | 7.78 | 12.80 | 1.64 | 0.27 | - | 12.45 | 0.97 |
| 0.37 | 7.35 | 12.52 | 1.70 | - | 7.59 | 13.21 | 1.74 |
| 0.17 | 9.57 | 11.95 | 1.25 | 0.10 | 8.80 | 10.69 | 1.22 |
| 0.13 | - | - | - | 0.07 | - | - | - |
| 0.27 | 7.71 | 13.07 | 1.69 | 0.00 | - | - | - |
| 0.10 | 9.41 | 12.81 | 1.36 | 0.00 | 10.51 | 13.38 | 1.27 |
| 0.13 | - | - | - | 0.17 | 10.07 | 12.66 | 1.26 |
| 0.53 | - | - | - | 0.17 | 8.23 | 12.74 | 1.55 |
| 0.17 | 8.67 | 13.24 | 1.53 | 0.03 | 9.98 | 13.77 | 1.38 |
| 0.80 | - | - | - | 0.23 | 7.05 | 11.77 | 1.67 |
| 0.33 | - | - | - | 0.00 | 6.45 | 11.26 | 1.75 |
| 0.20 | - | - | - | 0.24 | 7.26 | 11.06 | 1.52 |
| 0.07 | 9.86 | 12.47 | 1.26 | - | - | - | - |
| - | - | - | - | - | - | - | - |
| 0.00 | 7.84 | 13.15 | 1.68 | 0.00 | 8.30 | 13.74 | 1.66 |
| 0.17 | - | - | - | 0.10 | 7.64 | 11.61 | 1.52 |
| 0.13 | 8.63 | 12.23 | 1.42 | 0.10 | - | - | - |
| 0.60 | - | - | - | 0.30 | 7.16 | 12.01 | 1.68 |
| 0.13 | 7.28 | 12.43 | 1.71 | 0.43 | 7.75 | 12.90 | 1.67 |
| 0.41 | - | - | - | 0.17 | 7.57 | 11.68 | 1.54 |
| 0.27 | 8.56 | 11.17 | 1.30 | 0.27 | 9.22 | 10.99 | 1.19 |
| 0.03 | 8.05 | 11.25 | 1.40 | 0.07 | 8.15 | 11.85 | 1.46 |
| 0.13 | 7.19 | 12.14 | 1.69 | - | - | - | - |
| 0.30 | - | - | - | 0.07 | - | - | - |
| 0.79 | 7.53 | 11.23 | 1.49 | 0.07 | - | - | - |
| 0.60 | 8.06 | 12.06 | 1.50 | 0.57 | 7.18 | 12.32 | 1.72 |
| 0.07 | 7.81 | 11.02 | 1.41 | 0.13 | - | - | - |
| 0.40 | 7.28 | 11.61 | 1.59 | 0.70 | - | - | - |
| 0.43 | 8.60 | 11.49 | 1.34 | 0.13 | 6.94 | 11.10 | 1.60 |
| 0.17 | 8.68 | 12.46 | 1.43 | 0.00 | 8.36 | 12.20 | 1.46 |
| 0.10 | 8.21 | 10.12 | 1.23 | 0.03 | 9.58 | 11.51 | 1.20 |
| 0.03 | 9.57 | 11.48 | 1.20 | - | - | - | - |
| 0.27 | 7.86 | 11.06 | 1.41 | 0.20 | 8.00 | 11.38 | 1.42 |
| 0.50 | - | - | - | - | - | - | - |
| 0.03 | 9.43 | 12.80 | 1.36 | 0.00 | 8.88 | 13.31 | 1.50 |
| 0.17 | - | - | - | - | - | - | - |
| 0.13 | 7.88 | 11.93 | 1.51 | 0.27 | 7.43 | 12.11 | 1.63 |
| 0.17 | 7.77 | 11.50 | 1.48 | 0.13 | 7.07 | 11.73 | 1.66 |
| 0.43 | - | - | - | 0.53 | 7.86 | 10.95 | 1.39 |
| 0.20 | - | 12.08 | 1.01 | 0.38 | 9.76 | 12.43 | 1.27 |
| 0.00 | 8.62 | 12.65 | 1.47 | - | - | - | - |
| 0.63 | 6.95 | 11.73 | 1.69 | 0.60 | 8.94 | 11.70 | 1.31 |
| 0.20 | 8.96 | 12.37 | 1.38 | 0.40 | 8.41 | 12.33 | 1.47 |
| 0.10 | 8.28 | 12.59 | 1.52 | 0.27 | 8.06 | 12.76 | 1.58 |
| 0.13 | 9.23 | 11.93 | 1.29 | 0.37 | 6.66 | 12.40 | 1.86 |
| 0.73 | 6.65 | 10.97 | 1.65 | 0.50 | 8.03 | 11.76 | 1.46 |
